# First detection of *Hedgehog coronavirus 1* in Poland

**DOI:** 10.1038/s41598-022-06432-z

**Published:** 2022-02-11

**Authors:** Małgorzata Pomorska-Mól, Jakub J. Ruszkowski, Maciej Gogulski, Katarzyna Domanska-Blicharz

**Affiliations:** 1 Department of Preclinical Sciences and Infectious Diseases, Poznan University of Life Sciences, ul. Wołyńska 35, 60-637 Poznan, Poland; 2 Department of Animal Anatomy, Poznan University of Life Sciences, ul. Wojska Polskiego 71C, 60-625 Poznan, Poland; 3 University Centre for Veterinary Medicine, Szydłowska 43, 60-656 Poznan, Poland; 4 Department of Poultry Diseases, National Veterinary Research Institute, Al. Partyzantów 57, 24-100 Puławy, Poland

**Keywords:** Microbiology, Molecular biology

## Abstract

Hedgehogs are common in the majority of European countries and are known to host various pathogens, including viruses. The recent discovery of MERS-related coronaviruses (CoVs) in hedgehogs from Germany, France, the UK, China, and Italy suggests that hedgehogs may represent a wild reservoir of betacoronaviruses. This study reports the first detection and characterization of novel betacoronovirus, subgenus *Merbecovirus* in wild hedgehogs in Poland. The CoV RNA was detected in 10 out of 40 hedgehogs’ rectal swabs and in 1 out of 18 samples of the lung. No viral RNA was identified in the duodenum and kidney. There was no significant relationship between clinical status, gender, hedgehogs' age, and coronaviral RNA detection. Phylogenetic analysis showed that CoVs detected in our study grouped together with other representatives of *Hedgehog coronavirus 1* species identified in Western Europe. Our findings provide further evidence that hedgehogs are a natural reservoir of *Merbecovirus*. Considering the high mutation rate of CoVs and their potential for crossing interspecies barriers, the proper management of hedgehogs admitted to wildlife rehabilitation centres is needed. It cannot be excluded that merbecovirus strains detected in hedgehogs may recombine with other CoVs leading to new viruses with potential for interspecies transmission.

## Introduction

European hedgehogs (*Erinaceus europaeus*) and Northern white-breasted hedgehogs (*Erinaceus roumanicus*) are small nocturnal, insectivorous mammals that are active from April to September and hibernates from October to March, widely spread in Europe^1^. They feed primarily on invertebrates such as beetles, earthworms, and molluscs but also on pet food that is frequently found in gardens and shared with dogs and cats^1,2^. Initially, those species lived in semi-open rural areas or forests with many trees and lush vegetation to provide hiding places and nesting material^3^. With increasing urbanisation, hedgehogs are becoming more synanthropic and live in the majority of European villages and urban areas nowadays. Those habitats provide more food sources, daily nest sites, and protection from badgers (*Meles meles),* the most dangerous natural predators for hedgehogs^4^. Living in parks, backyard gardens, and other urban green areas exposes them to various human-related risks, such as traffic accidents, poisoning from multiple chemicals used in urban green spaces, and attacks from stray cats.

The increasing number of hedgehogs, especially in gardens and city parks, influence animal-human contact frequency. People trying to help hedgehogs may unwittingly expose themselves to various pathogens, including zoonotic ones. Hedgehogs are an essential vector of many zoonotic pathogens, including bacterial: *Salmonella* sp., *Mycobacterium* sp., Methicillin-Resistant *Staphylococcus aureus*, *Leptospira* spp^5–11^ and viral ones: tick-borne encephalitis virus (TBEV); Severe fever with thrombocytopenia syndrome virus (SFTSV)^12,13^. The recent discovery of MERS-related CoVs in hedgehogs from Germany^14^, France^15^, the UK^16^, China^17^, and Italy^2^ suggests that hedgehogs may represent a wild reservoir of CoVs.

Coronaviruses are enveloped positive-sense RNA viruses. They belong to the *Coronaviridae* family, order *Nidovirales*^18,19^. CoVs are pathogenic for mammals and birds, in which they cause infections manifested by a range of clinical signs (from respiratory, gastrointestinal, and nervous systems) and subclinical infections^18,20^. Their propensity to recombine allows them to easily transmit and adapt to new hosts^18,19^. Four genera of CoV have be identified to date: *Alphacoronavirus*, *Betacoronavirus*, *Gammacoronavirus* and *Deltacoronavirus*^21^. The *Betacoronavirus* genus of mammal-infecting viruses includes three subgenera (*Sarbecovirus*, *Embecovirus,* and *Merbecovirus*)^22^. The discovery of Erinaceus CoVs (EriCoVs) belonging to *Betacoronavirus* (subgenus *Merbecovirus*) in hedgehogs from various parts of the world^2,14–17^ may indicate their potential role as reservoirs and/or vectors of these CoVs. The main representative of *Merbecovirus* subgenus, MERS-CoV, has been proven to be zoonotic and pathogenic to humans^23^. It has been shown that merbecoviruses undergo recombination and show significant genetic variation^22^. The epidemiological, biological, and virological characteristics of CoVs, mainly based on Spike-protein plasticity, suggest species barriers to infection may be easily crossed^24,25^. Thus, CoVs identified in hedgehogs may pose a potential risk to humans, especially as there is increasing contact between hedgehogs and humans.

This study aimed to assess the presence of coronaviruses in hedgehogs in an urban area in Poland.

## Results

Twenty-six juvenile (65%) and 14 (35%) adult animals were included in the study. 67.5% were females (27/40), while 32.5% were males (13/40). The mean body weight of juvenile animals was 264.42 ± 104.81 (range 83–467 g), while in the adult group, it reaches 740.07 ± 200.12 (490–1150 g). The presence of coronavirus (CoV) RNA was detected in 10 out of 40 hedgehogs’ rectal swabs (25%). Additionally, when examining the organs of animals, coronaviral RNA was detected in 1 out of 18 samples of the lung (5.55%), and it was from a hedgehog previously identified as swab-positive. No viral RNA was identified in the duodenum and kidney collected from hedgehogs*.* In investigated group, the CoV presence was confirmed in 25% of samples (95% CI: 14.2–40.2). Four adult and two young hedgehogs were found to be underweight; the weight of the other animals was normal. One of ten CoV RNA positive hedgehogs was diagnosed with underweight, but the other animals were in normal condition. An equal number (n = 20) of clinically healthy and sick animals were used in this study (detailed characteristics are presented in Table 2). Most animals (70%) in which the CoV was detected were clinically healthy. The remaining positive hedgehogs were diagnosed with fractures (2/3) and severe ectoparasite infestation (1/3). Eighty % of positive hedgehogs were juveniles, while only 20% were adults, noting that the number of juveniles in our study was almost double that of adults. Females represented 90% (9/10) of positive animals. There was no significant relationship between clinical status, gender, age, and detection of hedgehog coronaviruses (Table 1).

**Table 1:** Detection of CoV RNA among hedgehog split into risk factors.

Risk factor	Positive/tested^a^	% Positive	*p*	OR (95% CI)
**Health status**			0.2733	0.328 (0.071–1.518)
Sick	3/20	15.00		
Clinically healthy	7/20	35.00		
**Gender**			0.1238	6.00 (0.671–53.685)
Female	9/27	33.33		
Male	1/13	7.69		
**Age**			0.4456	2.67 (0.481–14.789)
Juvenile	8/26	30.77		
Adult	2/14	14.28		

*p* value determined by two-sided Fisher’s exact test; *p* ≤ 0.05 considered significant.

*OR* Odds ratio, *95% CI* 95% confidence interval.

^a^Number of positive animals concerning all tested animals.

The odds ratio calculated for gender indicates a 6 times higher (95% CI 0.671–53.685) likelihood of having positive results in females compared to the males. After controlling for age, the odds of being bCoV RNA positive was 2.67 times higher in juvenile animals (95% CI 0.481–14.789).

### Phylogenetic analysis

**Figure 1:**
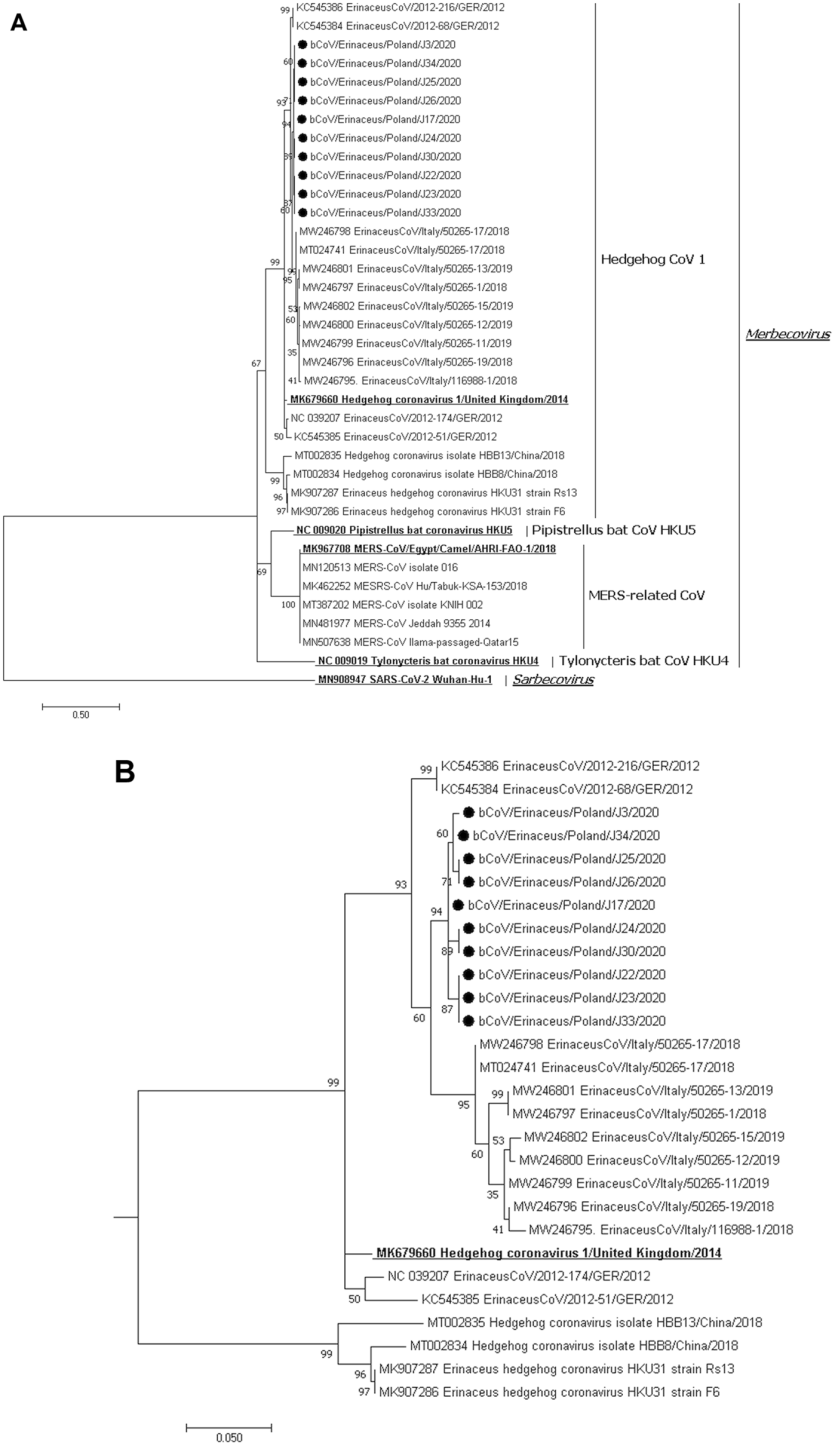
Phylogenetic analysis of betacoronaviruses based on the replicase gene fragment. (**A**) The tree constructed for 37 betacoronaviruses: 10 strains identified in hedgehogs in Poland (marked with a dot), 27 from GenBank including 5 reference strains (written in bold and underlined) representing ratified four species of the *Merbecovirus* subgenus, and fifth strain representing the only species of *Sarbecovirus* subgenus as the outgroup. (**B**) Separate subtree of betacoronaviruses of the *Hedgehog coronavirus 1* species. The tree was constructed using MEGA 7 using the maximum likelihood method based on the T92 + G + I model and 1000 bootstrap replicates (bootstrap values shown on the tree).

Topology of the phylogenetic tree based on the obtained sequence fragments of the RdRp gene revealed that ten of the positive hedgehogs resulted in being infected with merbecoviruses (Fig. 1A). Phylogenetic analysis showed that CoVs from Polish hedgehogs grouped together with other representatives of *Hedgehog coronavirus 1* (HedCoV1)*,* species identified in Western Europe. All 10 Polish hedgehog CoV strains were grouped within the same subgroup (Fig. 1B). The alignment of sequences of detected CoV strains showed identity between 98.1 and 100% and possessed the highest similarity (96.3–97.0%) with the viruses from German hedgehogs of subgroup 1 (GenBank numbers KC545384, KC545386). They also revealed high nt sequence similarity with Italian (95.4–96.8%) and German strains of subgroup 2 (93.5–95.0%) (MW246795-802, MT024741, and NC039207, KC545385, respectively). The nt homology of Polish hedgehog CoVs to strains sampled in Great Britain (MK679660) and China (MT002834-35, MK907286-87) were 92.8–93.3% and 83.5–84.7%, respectively.

## Discussion

Hedgehogs have been indicated as a possible wild reservoir of emerging CoVs with potential public health implications^14^. This study confirmed the presence of betacoronavirus (bCoV), subgenus *Merbecovirus* in hedgehogs in Poland for the first time. Our preliminary results suggest that CoVs are quite common in hedgehogs, while 25% of animals tested in our study were CoVs positive (95% CI: 14.2–40.2). The CoV RNA has also been confirmed in 1 out of 18 samples of the lung. No viral RNA was identified in other samples tested (duodenum and kidney). The presence of bCoVs in hedgehogs has been reported previously in other European countries: France^15^, Germany^14^, Italy^2^, Great Britain^16^, but their prevalence were generally higher than in our study (Germany 58.9%, France 50%, Italy 58%). In one study from Great Britain, the detected prevalence was lower (10.8%). However, it should be remembered that the presence of different viruses, including CoVs, may depend on many factors, such as sampling season, sample handling, and most of all, the detection method used^26^. In hedgehogs, additional variability in their prevalence may be caused by the fact that they hibernate, becoming inactive for a few months. After this period, their metabolism does not immediately reach the normative one. It could be expected that viruses in such hedgehog state replicate slowly and are not shed/shed in lower quantity than in the active state. However, the relatively high CoV RNA occurrence in hedgehogs included in our research may suggest that they play an important role as a wild reservoir of CoVs.

In accordance with previous reports, we did not observe any correlation between CoVs positivity and the health condition of animals^2,14,16^. CoVs positive hedgehogs did not reveal the clinical signs of CoV infection. One of ten CoV RNA positive hedgehogs was diagnosed with underweight, but the other animals were in good condition. *Coronaviridae* family appear to include viruses, a few of which cause disease. The number of detected microorganisms that are not correlated with any disease has increased significantly with the widespread use of molecular diagnostic tools. This also applies to coronaviruses detected in, i.e. bats or wild birds^27,28^. On the other hand, they could be opportunistic pathogens, the virulence of which is revealed under unfavourable conditions or in co-infections with other pathogens. In the present study, there was also no significant relationship between gender and age and detection of hedgehog CoVs, but juveniles and females had a higher likelihood of having positive results compared to the adult and males. It could be related to observing a much stronger amplification of CoV RNA observed in juvenile and lactating female bats of the species *Myotis* sp.^29^. In addition, juveniles are susceptible to all infections and only develop immunity over the years due to contact with different infectious agents. The CoVs detected in hedgehogs in our study were highly homologous to each other and similar to others from Europe in the compared fragment of the genome (93.5 to 97.0% homology). They differed most from the CoVs detected in *Erinaceus amurensis* in China (83.5 to 84.7% homology). It is unknown if these differences in the studied genome fragment sequence result from geographical segregation or reflect “species-dependent” grouping. However, it should be noted that in our study, hedgehogs came from a relatively small area. On the other hand, when the same genome fragment was compared, a “species-dependent” grouping of gamma- and deltacoronaviruses identified in wild birds was observed^30^.

The progenitors of the three coronaviruses (SARS-CoV-1, SARS-CoV-2, MERS-CoV), which caused the most important epidemics in humans, seem to originate from bats^22,24^. During passaging in different intermediate organisms, these progenitors evolving through mutation and/or recombination gained genetic features that enabled infection and transmission between humans^21,22^. Perfect conditions for the emergence of viruses with new characteristics are during co-infection with two or more viruses when homologous recombination occurs^18,20^. Such emerging viruses could acquire the possibility of host switch, more efficacious replication, and/or more virulent. That is why it is important to monitor the prevalence of CoVs in animals already known as hosts for CoVs (e.g., bats, hedgehogs) and other yet unexplored. Such studies have recently identified novel sarbecovirus in a British horseshoe bat, confirming that these viruses are distributed not only in Asian rhinolophids and revealed that the opportunities for emerging zoonoses are persistent and globally threatening^31^. As bats and hedgehogs are phylogenetically closely related and both are insectivorous animals^17,32^, future studies on the circulation of CoVs in these species may shed light on the diversity and evolution of merbecoviruses.

Considering all the above aspects, the proper management of hedgehogs admitted to wildlife rehabilitation centres, especially multi-species, seems extremely important. At present, it cannot be excluded that *Merbecovirus* strains detected in hedgehogs may recombine with other CoVs (e.g., from bats or other animals kept in such places), leading to new viruses with potential for interspecies transmission (e.g., from bats to humans). It should also be remembered that these animals usually return to their original areas of distribution after a period of rehabilitation/treatment or rearing, potentially spreading these possibly newly emerged viruses into the environment.

Future studies involving the higher number of hedgehogs from various regions of Poland and other wildlife species are planned to verify the results presented here. It also seems important to obtain whole-genome sequences of the identified merbecoviruses, and such attempts are currently underway.

## Materials and methods

### Sample collection

Since hedgehogs are protected by Polish law (Regulation of the Minister of the Environment of 16 December 2016, on the protection of animal species (Journal of Laws, item 2183) and (Journal of Laws 2020, item 26), all procedures were approved and carried out in accordance with the appropriate regulations and permits (Regional Directorate for Environmental Protection in Poznan (Poland): WPN-II.6401.366.2020.TE). We followed the ARRIVE guidelines during the study.

Rectal swabs were collected from 40 hedgehogs brought to the Wildlife Rehabilitation Centre (WRC) in Poznan after being found sick, injured, or too young to survive on their own for various diagnostic purposes. All hedgehogs were found on the urban area of the city of Poznan (16° 55′ E; 52° 25′ N), Wielkopolskie Voivodeship, Poland. When admitted to the WRC, hedgehogs were kept in isolation until sample collection (within 12 h of admission) to reduce the possibility of nosocomial infections. Between August 2020 and December 2020, the 40 individual rectal swabs were collected using swabs with transport medium (UTM: Viral Transport, COPAN Diagnostics, USA) and stored at − 80 °C until laboratory examinations. In addition, samples of duodenum, lung, and kidney from 18 hedgehogs that were dead at admission died or were euthanised (with the use of xylazine and ketamine administered intramuscularly followed by intravenous pentobarbital administration) for ethical reasons (spine, bone fractures) during their stay at WRC, were collected and stored at − 80 °C until analyses. The following information for each hedgehog was recorded: delivery date, gender, body weight, age, health status. Detailed characteristic of animals used in the study is presented in Table 2.

**Table 2:** Demographic information and health status of hedgehogs included in the study.

Delivery date	Sex	Age	Body weight (g)	Clinical condition on admission, diagnosis	CoV status	GenBank number
08.2020	F	juv	335	Weak, spinal fracture, euthanasia	−	
08.2020	F	juv	309	Weak, jaw fracture, died in the WRC	−	
08.2020	M	ad	781	Weakness due to infestation of external parasites	+	MZ605015
08.2020	F	ad	490	Abscess (lungs), mucocele, weakness, uterus with ampullary changes	−	
08.2020	F	juv	225	Mucocele, wounds on back, died in the WRC	−	
08.2020	M	ad	507	Extensive back wound, extreme cachexia, mucocele, died in the WRC	−	
08.2020	M	ad	780	Extreme weakness, lots of ticks and fleas	−	
08.2020	F	ad	574	Fracture of the spine, pelvis and femur, euthanasia	−	
08.2020	M	juv	246	Urinary tract haemorrhage, femur fracture, euthanasia	−	
08.2020	F	ad	1150	Clinically healthy, pregnant female	−	
08.2020	M	juv	400	Clinically healthy	−	
08.2020	M	juv	384	Clinically healthy	−	
08.2020	M	juv	150	Clinically healthy	−	
08.2020	F	juv	135	Clinically healthy	−	
08.2020	M	juv	148	Clinically healthy	−	
08.2020	M	ad	540	Extremely weak, died in the WRC	−	
09.2020	F	ad	777	Spinal fracture, skinned left pelvic limb, euthanasia	+	MZ605016
09.2020	F	juv	136	Dyspnoea	−	
09.2020	F	juv	160	Clinically healthy	−	
09.2020	F	ad	1010	Weak, died up to 24 h after admission; hepato- and splenomegaly	−	
09.2020	F	juv	289	Clinically healthy	+	MZ605017
09.2020	F	juv	354	Clinically healthy	−	
09.2020	F	juv	419	Clinically healthy	+	MZ605018
09.2020	F	juv	292	Clinically healthy	+	MZ605019
09.2020	F	juv	83	Clinically healthy	+	MZ605024
10.2020	F	juv	197	Clinically healthy	−	
10.2020	F	juv	103	Clinically healthy	+	MZ605020
10.2020	F	juv	211	Clinically healthy	−	
10.2020	F	juv	322	Clinically healthy	−	
10.2020	F	juv	249	Clinically healthy	+	MZ605021
10.2020	F	ad	708	Clinically healthy, slightly weakened	−	
10.2020	F	ad	817	Weak	−	
10.2020	F	juv	292	Clinically healthy	+	MZ605022
10.2020	F	juv	277	Weak, fracture of right femur, died after admission	+	MZ605023
10.2020	F	juv	302	Weak, spinal fracture, euthanasia	−	
10.2020	F	ad	897	Found dead	−	
10.2020	M	juv	467	Clinically healthy	−	
10.2020	M	ad	510	Dehydrated, diarrhoea, mucocele, severe suppurative arthritis	−	
11.2020	M	juv	390	Trauma of the left pelvic limb, necrosis of the distal part of the limb	−	
12.2020	F	ad	820	Found dead	−	

*ad* Adult, *juv* Juvenile, + CoV-positive individuals, − CoV-negative individuals.

### Molecular detection of coronavirus RNA

Hedgehogs’ tissues were homogenised in a sterile phosphate-buffered saline (Biomed, Lublin, Poland), obtained suspensions (10% w/v) were clarified by centrifugation (15 min at 3000×g and 4 °C and the supernatant used for RNA isolation. All swab transport media were also centrifuged before the process of RNA isolation. The RNA from 200 ul of the obtained fluids was extracted using an RNeasy Mini Kit (Qiagen, Hilden, Germany) according to the manufacturer’s instructions. The samples were tested for CoV presence using an RT-PCR assay in a nested configuration using primers with a few degenerate nucleotides^33^. The assay amplifies a 555-nucleotide gene fragment of the non-structural protein 12 RNA dependent RNA polymerase (nsp12 RdRp), one of the components of viral replicase machinery present in all coronaviruses of mammalian and avian origin. All reactions were performed in a final volume of 25 µl. For RNA transcription and in the first step of DNA amplification, the One-Step RT-PCR kit (Qiagen, Hilden, Germany) was used. The Platinum Taq DNA Polymerase kit (Invitrogen, Carlsbad, USA) and the PCR product in dilution of 1:5 (v/v) were used in the second step. The vaccine IBV 4/91 strain (Nobilis IB 4/91, MSD Animal Health, USA) served as a positive control. The PCR-positive samples were identified using agarose gel electrophoresis. Obtained amplicons were sequenced in both directions using Sanger sequencing technology by Genomed Sp. z o.o. (Warsaw, Poland).

### Sequence and phylogenetic analysis

The forward and reverse nucleotide sequences were edited and aligned in the final consensus using Geneious v11.1.3 (Biomatters, Ltd., Auckland, New Zealand). They were then compared with sequences published in the GenBank database using the Basic Local Alignment Search Tool (BLAST) with the default parameters. Sequences with the highest homology were downloaded for further analysis. These sequences, together with 5 sequences of reference strains including 4 representing individual species from *Merbecovirus* subgenus (HedCoV1*, Middle East respiratory syndrome-related coronavirus, Pipistrellus bat coronavirus HKU, Tylonycteris bat coronavirus HKU4*), and the only one representing *Sarbecovirus* subgenus of the *Betacoronavirus* genus were then aligned using the MAFFT method in Geneious. The alignments were then exported to MEGA software, v7.0.26^34^. A maximum likelihood (ML) phylogenetic analysis was conducted using the best-fitting nucleotide substitution models. A bootstrap test including 1,000 replicates was performed for each resultant tree.

### Statistical analyses

The associations between CoV RNA detection in samples, demographic features (species, gender, age), and health status variables were estimated using Fisher’s exact test. The Wilson method for small n was used to calculate a 95% confidence interval (95% CI) for CoV RNA prevalence. All statistical analyses were performed in Statistica13.3 software (Tibco, USA).

### Ethics declarations

All procedures were approved and carried out in accordance with the appropriate regulations and permits (Regional Directorate for Environmental Protection in Poznan (Poland): WPN-II.6401.366.2020.TE) and carried out according to the guidelines of the European Council Directive 86/609/EEC dated November 1986. The reported study complies with the ARRIVE guidelines.

## Data Availability

The viral sequences obtained in this study were deposited at NCBI Genbank, accession numbers: MZ605015-MZ605024. Other relevant data analysed during this study are included in this manuscript.
